# Existence of zero-order meromorphic solutions of certain *q*-difference equations

**DOI:** 10.1186/s13660-018-1790-z

**Published:** 2018-08-20

**Authors:** Yunfei Du, Zongsheng Gao, Jilong Zhang, Ming Zhao

**Affiliations:** 1 LMIB-School of Mathematics and Systems Science, Beihang University, Beijing, China; 2 School of Science, China University of Geosciences (Beijing), Beijing, China

**Keywords:** 39B32, 34M05, 30D35, Painlevé equations, *q*-Difference, Meromorphic solution

## Abstract

In this paper, we consider the *q*-difference equation

$$ \bigl(f(qz)+f(z)\bigr) \bigl(f(z)+f(z/q)\bigr)=R(z,f), $$

where $R(z,f)$ is rational in *f* and meromorphic in *z*. It shows that if the above equation assumes an admissible zero-order meromorphic solution $f(z)$, then either $f(z)$ is a solution of a *q*-difference Riccati equation or the coefficients satisfy some conditions.

## Introduction

In this paper, we use the basic notions of Nevanlinna theory [1–4] such as the characteristic function $T(r,f)$, counting function $N(r,f)$, and proximity function $m(r,f)$. We also use $S(r,f)$ to denote any quantity satisfying $S(r,f)=o(T(r,f))$ as $r\rightarrow \infty $, possibly outside a set with finite logarithmic measure and $S(f)$ to denote the field of small functions with respect to *f*, which is defined as $S(f)=\{\alpha \text{ meromorphic} : T(r, \alpha )=S(r,f)\}$.

In what follows, we use the short notation $\bar{f}\equiv f(z+1)$ and $\underline{f}\equiv f(z-1)$. A meromorphic solution *f* of a difference equation is called *admissible* if all the coefficients of the equation are in $S(f)$. In particular, if the coefficients are rational, then an admissible solution must be transcendental, and if an admissible solution is rational, then the coefficients must be constants.

An ordinary differential equation is said to possess the Painlevé property if all of its solutions are single-valued about all movable singularities, see [5]. In 1895–1910, Painlevé [6, 7], Fuchs [8], and Gambier [9] completed substantial classification work, which comprised sieving through a large class of second-order differential equations by making use of a criterion proposed by Picard [10], now known as the Painlevé property. Painlevé and his colleagues discovered six new equations, later named Painlevé equations, which were not solvable in terms of known functions. Actually, the Painlevé equations are six nonlinear ordinary differential equations denoted traditionally by $P_{I}$, $P _{II}$, … , $P_{VI}$.

Painlevé equations is a fascinating subject in mathematics, they possess many special features [11]. One of them is that, given a solution of a Painlevé equation $(P_{II}, \ldots , P_{VI})$ with a choice of some parameter, a special method based on Bäcklund transformations can be used for deriving a new solution with a different value of the parameter, either for the same Painlevé equation or for another. Symmetry is a word used frequently to refer to a mechanism of constructing new solutions by transformation. Specially, Painlevé equation appeared in many applications and fields such as hydrodynamics, plasma physics, nonlinear optics, solid state physics, etc.

As for the difference type Painlevé equation, many scholars have focused on it and given many useful results [12–22]. In particular, Ablowitz, Halburd, and Herbst [23] studied the following Painlevé difference equation:

$$ \bar{f}\star \underline{f}=R(z,f), $$

where *R* is rational in both of its arguments, ⋆ stands for either the addition or the multiplication. They proved that the existence of a nonrational meromorphic solution of finite order implies $\deg_{f}R\leq 2$. This class of equations contains many integrable equations called difference Painlevé I–III equations.

Halburd and Korhonen [24] considered the equation

1 $$ \bar{f}+\underline{f}=R(z,f), $$

where $R(z,f)$ is rational in *f* and meromorphic in *z*. And they proved if (1) admits an *admissible* finite order meromorphic solution, then either $f(z)$ satisfies a difference Riccati equation or (1) can be transformed into difference Painlevé I, II equations or a list of linear difference equations. The work on the family $\bar{f}\underline{f}=R(z,f)$, which includes the so-called difference Painlevé III, was initiated in [25].

Ronkainen [21] researched the family

2 $$ (\bar{f}f-1) (f\underline{f}-1)=R(z,f), $$

which includes the difference Painlevé V equations.

The family of following equations [26], which includes the difference Painlevé IV

3 $$ (\bar{f}+f) (f+\underline{f})=R(z,f) $$

with constant coefficients, was studied by Grammaticos *et al.* [27]. Furthermore, Wen [28] and Zhang [18] researched (3) with $R(z,f)$ rational in *f* and meromorphic in *z* from a different aspect.

The aim of this paper is to investigate the second order *q*-difference equation

4 $$ \bigl(f(qz)+f(z)\bigr) \bigl(f(z)+f(z/q)\bigr)=R(z,f), $$

where $|q|\notin \{0,1\}$ and $R(z,f)$ is rational in *f* and meromorphic in *z*. We first discuss the possible degrees of $R(z,f)$, then fix the degree. We prove that if the equation admits an admissible zero-order meromorphic solution $f(z)$, then either $f(z)$ is a solution of some *q*-difference Riccati equations or the coefficients of (4) satisfy some conditions. Our research is a generalization of the related results [18, 28]. Actually, we extend difference Painlevé IV equations to a *q*-difference form, which is a supplement and completeness for studying Painlevé equations.

## Some lemmas

We introduce some lemmas for the proofs of our theorems in this section. The logarithmic derivative lemma [2] plays an important role in difference equations. As for *q*-difference equation, Barnett et al. [19] gave the analogue of the logarithmic derivative lemma, we recall it as follows.

### Lemma 2.1

*Let* $f(z)$ *be a non*-*constant zero*-*order meromorphic function*, *and* $q\in C \setminus \{0\}$. *Then*

$$ m \biggl( r, \frac{f(qz)}{f(z)} \biggr) =o\bigl(T(r, f)\bigr) $$

*on a set of logarithmic density* 1.

### Lemma 2.2

([19])

*Let* $f(z)$ *be a non*-*constant meromorphic solution with zero*-*order of the equation* $P(z,f)=0$, *where* $P(z,f)$ *is a q*-*difference polynomial in* $f(z)$. *If* $P(z,a)\not \equiv 0 $ *for a meromorphic function* $a\in S(f)$, *then*

$$ m \biggl( r,\frac{1}{f-a} \biggr) =S(r,f) $$

*on a set of logarithmic measure density* 1.

### Lemma 2.3

([14])

*Let* $f(z)$ *be a zero*-*order meromorphic function*, *and* $q\in C\setminus \{0\}$. *Then*

$$ \begin{gathered} T\bigl(r,f(qz)\bigr)=T\bigl(r,f(z)\bigr) \bigl(1+o(1)\bigr), \\ N\bigl(r,f(qz)\bigr)=N\bigl(r,f(z)\bigr) \bigl(1+o(1)\bigr) \end{gathered} $$

*on a set of lower logarithmic density* 1.

The next lemma is an essential result about the Nevanlinna characteristic and plays an important role in the difference equations.

### Lemma 2.4

([27])

*Let* *f*, *h*, *and* *g* *be three meromorphic functions*. *Then*

$$ T(r, fg+gh + hf )\leq T(r, f ) + T(r, g) + T(r, h) + O(1). $$

The following lemma, i.e., the Valiron–Mohon’ko identity [29, 30], is a useful tool in the theory of complex difference equations.

### Lemma 2.5

*Let* *f* *be a meromorphic function*. *Then*, *for all irreducible rational functions in f*,

$$ R(z,f)= \frac{P(z, f)}{Q(z,f)}=\frac{\sum_{i=0}^{p} a_{i}(z)f^{i}}{ \sum_{j=0}^{q} b_{j}(z)f^{j}} $$

*with meromorphic coefficients* $a_{i}(z), b_{j}(z)\in S(f)$, *then the characteristic function of* $R(z, f(z))$ *satisfies*

$$ T\bigl(r, R(z,f)\bigr)=(\deg_{f}R)T(r,f)+S(r,f)=\max (p, q)T(r,f)+S(r,f). $$

## Main result

In this paper, we consider the following *q*-difference equation:

5 $$ \bigl( f(qz)+f(z) \bigr) \bigl( f(z)+f(z/q) \bigr) =R(z,f)= \frac{P(z,f)}{Q(z,f)}, $$

where $P(z,f)$, $Q(z,f)$ are irreducible polynomials in *f* with degrees *p* and *q*, respectively.

In the following, we discuss the possible degrees of $P(z,f)$ and $Q(z,f)$. Applying Lemma 2.3 yields

6 $$\begin{aligned} \deg_{f} (R)T(r,f) \leq & 2T\bigl(r,f(z)\bigr)+T\bigl(r,f(qz) \bigr)+T\bigl(r,f(z/q)\bigr)+S(r,f) \\ =& 4T(r,f)+S(r,f), \end{aligned}$$

which means $\deg_{f} (R)\leq 4$. Then, basing on Lemma 2.5 and equation (5), we have $p\leq 4$ and $q\leq 4$.

We rewrite (5) as

7 $$\begin{aligned} K(z,f)=:f(qz)f(z)+f(qz)f(z/q)+f(z)f(z/q)= \frac{P(z,f)-f^{2}(z)Q(z,f)}{Q(z,f)}. \end{aligned}$$

From Lemma 2.3 and Lemma 2.4, we have

8 $$\begin{aligned} \deg_{f} (K)T(r,f) \leq & T\bigl(r,f(z)\bigr)+T \bigl(r,f(qz)\bigr)+T\bigl(r,f(z/q)\bigr)+S(r,f) \\ =& 3T(r,f)+S(r,f), \end{aligned}$$

which implies $\deg_{f} (K)\leq 3$. Since $P(z,f)$ and $Q(z,f)$ are irreducible polynomials, then $P(z,f)-f^{2}(z)Q(z,f)$ and $Q(z,f)$ have no common factors, thus $p\leq 5$ and $q\leq 3$ follows.

Combining the above discussion, it may happen $p\leq 4$ and $q\leq 3$. If $q=3$, the degree of $P(z,f)-f^{2}(z)Q(z,f)$ will be 5, which contradicts $\deg_{f} (K)\leq 3$. Hence, it must have $p\leq 4$ and $q\leq 2$.

If $q=2$, it follows that the degree of $P(z,f)-f^{2}(z)Q(z,f)$ cannot be more than 3, that $q=4$, and the coefficients of the highest degree of $P(z,f)$ and $Q(z,f)$ are identical.

Therefore, we conclude the following result.

### Theorem 3.1

*If* $f(z)$ *is an admissible zero*-*order meromorphic solution of* (5), *where* $P(z,f)$, $Q(z,f)$ *are irreducible polynomials in* *f* *with degrees* *p* *and* *q*, *respectively*, *then we have* $p\leq 2$ *and* $q\leq 4$. *Particularly*, *one of the following holds*:

(i) *If* $q=0$, *then* $p\leq 2$;
(ii) *If* $q=1$, *then* $p\leq 3$;
(iii) *If* $q=2$, *then* $p=4$ *and the coefficients of the highest degree of* $P(z,f)$ *and* $Q(z,f)$ *are identical*.

### Proof

From the above discussion, we know that (ii) and (iii) hold. Next we give the proof of (i). Otherwise, if $q=0$ and $p=3$, then equation (5) can be rewritten as

9 $$\begin{aligned} \bigl( f(qz) +f(z) \bigr) \bigl( f(z) +f(z/q) \bigr) = a_{1}(z)f ^{3}(z) +a_{2}(z)f^{2}(z) + a_{3}(z) f(z) +a_{0}(z), \end{aligned}$$

where $a_{0}(z)$, $a_{1}(z)$, $a_{2}(z)$, and $a_{3}(z)$ are small functions to $f(z)$.

From equation (9), we have

10 $$\begin{aligned} 3T(r,f) +S(r,f) =& T\bigl(r, a_{1}(z)f^{3}(z) +\bigl(a_{2}(z)-1\bigr)f^{2}(z) + a _{3}(z) f(z) +a_{0}(z)\bigr) \\ =& T\bigl(r,f(qz)f(z) + f(z/q)f(z) + f(qz)f(z/q)\bigr) \\ =& m\bigl(r,f(qz)f(z) + f(z/q)f(z) + f(qz)f(z/q)\bigr) \\ &{}+ N\bigl(r,f(qz)f(z) + f(z/q)f(z) + f(qz)f(z/q)\bigr) \\ \leq & m \biggl( r,\frac{f(qz)f(z) + f(z/q)f(z) + f(qz)f(z/q)}{f ^{2}(z)} \biggr) \\ &{}+ m\bigl(r,f^{2}(z)\bigr) + N\bigl(r,f(qz)f(z) + f(z/q)f(z) + f(qz)f(z/q)\bigr) \\ =& 2m\bigl(r,f(z)\bigr) + 3N\bigl(r,f(z)\bigr)+S(r,f), \end{aligned}$$

which implies $N(r,f)=T(r,f)+S(r,f)$, so $f(z)$ has infinitely many poles. Let $z_{0}$ be a pole of $f(z)$ with multiplicity $k_{0}$, and denote it by $f(z_{0})=\infty^{k_{0}}$. Then either there is a cancelation with a zero or pole of some of the coefficients in (9) or at least one of $f(qz_{0})=\infty^{k_{1}}$ and $f(z_{0}/q)=\infty^{k_{2}}$ holds. Since the coefficients are small functions to $f(z)$, we can always choose a pole of $f(z)$ in such a way that there is no cancelation with the coefficients. Thus we just consider the condition that at least one of $f(qz_{0})=\infty^{k_{1}}$ and $f(z_{0}/q)=\infty^{k_{2}}$ holds. Comparing the orders of poles on both sides of equation (9), we can get

$$ \max \{k_{1}, k_{2}\}\geq \frac{3}{2}k_{0}. $$

Without loss of generality, we assume $k_{1}\geq \frac{3}{2}k_{0}$. Putting *q*-shift to (9), we obtain

11 $$ \bigl( f\bigl(q^{2}z\bigr) + f(qz) \bigr) \bigl( f(qz) + f(z) \bigr) = a_{1}(qz)f^{3}(qz) +a_{2}(qz)f^{2}(qz) +a_{3}(qz) f(qz) +a_{0}(qz), $$

then $f(q^{2}z)=\infty^{2k_{1}}$ follows. Continuing the iteration to equation (9), we will get a sequence of poles $f(q^{n}z)= \infty^{2^{(n-1)}k_{1}}$. Thus, $N(r,f)$ is at least exponential growth, which contradicts that $f(z)$ is of zero-order. Therefore, $p\leq 2$. This completes the proof of (i). □

In the following, we discuss the cases $p=4$ and $q=2$. Actually, we obtain the result as follows.

### Theorem 3.2

*If* $f(z)$ *is an admissible zero*-*order meromorphic solution of the equation*

12 $$ \bigl( f(qz)+f(z) \bigr) \bigl( f(z)+f(z/q) \bigr) = \frac{c(z)\prod_{i=1} ^{4}(f(z)-a_{i}(z))}{(f(z)-b_{1}(z))(f(z)-b_{2}(z))}, $$

*where the coefficients* $a_{i}(z)$ ($i=1,\ldots,4$), $b_{j}(z)$ ($j=1,2$) *are distinct meromorphic functions*, $c(z)\not \equiv 0$ *and* $a_{i}(z), b _{j}(z), c(z)\in S(f) $, *then* $c(z)\equiv 1$. *Furthermore*, *if* $|q|\notin \{0,1\}$, *then either* $f(z)$ *satisfies a* *q*-*difference Riccati equation*

$$ w(qz)=\frac{\alpha (z)f(z)+\beta (z)}{f(z)+\gamma (z)} $$

*where* $\alpha (z), \beta (z), \gamma (z) \in S(f)$, *or one of the following holds*:

- $b_{1}(q^{2}z)=b_{1}(z)$ *and* $b_{2}(q^{2}z)=b_{2}(z)$;
- $b_{1}(q^{2}z)=b_{2}(z)$ *and* $b_{2}(q^{2}z)=b_{1}(z)$.

### Proof

If $c(z)\not \equiv 1$, then equation (12) can be rewritten as

13 $$ f(qz)f(z)+f(z)f(z/q)+f(qz)f(z/q)=\frac{c\prod_{i=1}^{4}(f-a _{i})-f^{2}\prod_{j=1}^{2}(f-b_{i})}{(f-b_{1})(f-b_{2})}. $$

Applying the Valiron–Mohon’ko theorem and Lemma 2.4 to equation (13) yields

14 $$ 4T(r,f)\leq 3T(r,f)+S(r,f), $$

which is impossible. Thus $c(z)\equiv 1$, then equation (12) is changed into

15 $$ \bigl( f(qz)+f(z) \bigr) \bigl( f(z)+f(z/q) \bigr) = \frac{\prod_{i=1}^{4}(f(z)-a _{i}(z))}{(f(z)-b_{1}(z))(f(z)-b_{2}(z))}. $$

Suppose $f(z)$ is a zero-order transcendental meromorphic solution of equation (15) and denote

$$ P(z,f) = \bigl(f(qz)+f(z)\bigr) \bigl(f(z)+f(z/q)\bigr) \bigl(f(z)-b_{1}(z) \bigr) \bigl(f(z)-b_{2}(z)\bigr)- \prod_{i=1}^{4}(f(z)-a_{i}(z). $$

It follows from $b_{j} \not \equiv a_{i}$ that $P(z,b_{j})\not \equiv 0$, then combining Lemma 2.2 yields

16 $$ m \biggl( r, \frac{1}{f-b_{1}} \biggr) =S(r,f) \quad \text{and} \quad m \biggl( r, \frac{1}{f-b_{2}} \biggr) =S(r,f), $$

i.e.,

17 $$\begin{aligned} N \biggl( r, \frac{1}{f-b_{1}} \biggr) =T(r,f)+S(r,f) \quad \text{and} \quad N \biggl( r, \frac{1}{f-b_{2}} \biggr) =T(r,f)+S(r,f), \end{aligned}$$

which implies $f(z)-b_{j}(z)$ ($j=1,2$) has infinitely many zeros. Suppose $z_{0}$ satisfies $f(z_{0})-b_{j}(z_{0})=0$, that is to say, $f(z_{0})=b_{1}(z_{0})$ or $f(z_{0})=b_{2}(z_{0})$. From equation (15), we have two possibilities:

(I) $f(z_{0})=a_{i}(z_{0})$ ($i=1,2,3,4$);
(II) $f(qz_{0})=\infty$ or $f(z_{0}/q)=\infty $.

Let $A=\{z_{i} \in C: i \in N\}$ be the multi-set of zeros of

$$ f(z)-b_{1}(z)=0 \quad \text{and}\quad f(z)-b_{2}(z)=0 $$

satisfying (II).

In what follows, we adopt the notation $a_{\star }$ and $b_{\ast }$ to represent $a_{i}$ ($i=1,\ldots,4$) and $b_{j}$ ($j=1,2$), respectively.

Let $n_{A}(r,\frac{1}{f-b_{j}})$ ($j=1,2$) be the counting function of the multi-set $A \cap \{z \in C: |z|\leq r\}$ and $N_{A}(r,\frac{1}{f-b _{j}})$ ($j=1,2$) represents the integrated counting function. Similarly, we use $N_{I}$ to represent the corresponding integrated counting function which satisfies condition (I).

Next, we will prove that the zeros of $f(z_{0})=b_{1}(z_{0})$ and $f(z_{0})=b_{2}(z_{0})$ are “almost all” satisfying (II), i.e.,

18 $$ N_{A} \biggl( r, \frac{1}{f-b_{\ast }} \biggr) =N \biggl( r, \frac{1}{f-b_{ \ast }} \biggr) +S(r,f)=2T(r,f)+S(r,f). $$

Otherwise if there are more than $S(r,f)$ points $z'$ such that (I) holds, then these points satisfy $a_{\star }(z')=f(z')=b_{\ast }(z')$, which gives that

19 $$ S(r,f)< N_{I} \biggl( r,\frac{1}{a_{\star }-b_{\ast }} \biggr) . $$

Combining (19) and $a_{i}, b_{j} \in S(f)$ yields

$$ S(r,f)< N_{I} \biggl( r,\frac{1}{a_{\star }-b_{\ast }} \biggr) =S(r,f), $$

which is a contradiction. Thus, equation (18) is established.

Let *B* be the subset of *A*, in which all the points are such that

$$ f\bigl(q^{2}z_{0}\bigr)=b_{1} \bigl(q^{2}z_{0}\bigr)\quad \text{or}\quad f \bigl(q^{2}z_{0}\bigr)=b_{2}\bigl(q^{2}z _{0}\bigr), $$

and the corresponding integrated counting function is denoted by $N_{B} ( r, \frac{1}{f-b_{\ast }} ) $.

Furthermore, we denote

$$ \bar{q}=\max \biggl\{ \vert q \vert ,\frac{1}{ \vert q \vert } \biggr\} $$

and use

$$ N_{A\setminus B} \biggl( r, \frac{1}{f-b_{\ast }} \biggr) =N_{A} \biggl( r, \frac{1}{f-b _{\ast }} \biggr) -N_{B} \biggl( r, \frac{1}{f-b_{\ast }} \biggr) $$

to represent the integrated counting function for $z \in \{A\setminus B\}$.

Therefore, for each two points in $B \cap \{z \in C: |z|< r\}$, there is exactly one pole in the disc $\{z \in C: |z|\leq \bar{q}r\}$, which can be uniquely associated with them. Then we have

20 $$ N_{B}(r, f) \leq N_{B} \biggl( \bar{q}r, \frac{1}{f-b_{1}} \biggr) \leq N _{B}\bigl(\bar{q}^{2}r, f \bigr) $$

and

21 $$ N_{B}(r, f) \leq N_{B} \biggl( \bar{q}r, \frac{1}{f-b_{2}} \biggr) \leq N _{B}\bigl(\bar{q}^{2}r, f \bigr). $$

In combination with (20), (21), and Lemma 2.3, we obtain

22 $$ 2N_{B}(r, f)=N_{B} \biggl( r, \frac{1}{f-b_{\ast }} \biggr) +S(r,f). $$

Similarly, if there is one point in $A\setminus B \cap \{z\in C: |z| \leq r\}$, then there exists at least one pole in the disc $\{z \in C :|z|\leq \bar{q}r \}$. Therefore, we have

23 $$ N_{A\setminus B} \biggl( r,\frac{1}{f-b_{\ast }} \biggr) \leq N_{A\setminus B} ( \bar{q}r,f ) . $$

We proceed to proving that the points in *A* are “almost all” in *B*, that is,

24 $$\begin{aligned} N_{B} \biggl( r,\frac{1}{f-b_{\ast }} \biggr) =N_{A} \biggl( r,\frac{1}{f-b _{\ast }} \biggr) +S(r,f). \end{aligned}$$

Otherwise, if there exists some $0 \leq \alpha <1$ such that

25 $$ N_{B} \biggl( r,\frac{1}{f-b_{\ast }} \biggr) \leq \alpha N_{A} \biggl( r,\frac{1}{f-b _{\ast }} \biggr) +S(r,f), $$

then it follows from (18) and (22) that

26 $$ N_{B}(r,f)\leq \frac{\alpha }{2} N_{A} \biggl( r,\frac{1}{f-b_{\ast }} \biggr) +S(r,f)= \alpha T(r,f)+S(r,f). $$

Combining (18), (22), (23), (26), and Lemma 2.3 yields

27 $$\begin{aligned} 2T(r,f) =& N_{A} \biggl( r,\frac{1}{f-b_{\ast }} \biggr) +S(r,f) \\ =&N_{A\setminus B} \biggl( r,\frac{1}{f-b_{\ast }} \biggr) +N_{B} \biggl( r,\frac{1}{f-b _{\ast }} \biggr) +S(r,f) \\ \leq & 2N_{B}(\bar{q}r,f)+N_{A\setminus B}(\bar{q}r,f)+S(r,f) \\ \leq &N_{B}(\bar{q}r,f)+N_{A}(\bar{q}r,f)+S(r,f) \\ \leq &(1+\alpha )T(r,f)+S(r,f), \end{aligned}$$

which is impossible for $0\leq \alpha <1$. So assumption (25) cannot hold. Thus,

28 $$\begin{aligned} N_{B} \biggl( r,\frac{1}{f-b_{\ast }} \biggr) =N_{A} \biggl( r,\frac{1}{f-b _{\ast }} \biggr) +S(r,f)=2T(r,f)+S(r,f), \end{aligned}$$

which can be divided into the following four cases:

- $f(z_{0})=b_{1}(z_{0})$ and $f(q^{2}z_{0})=b_{2}(q^{2}z _{0})$;
- $f(z_{0})=b_{2}(z_{0})$ and $f(q^{2}z_{0})=b_{1}(q^{2}z _{0})$;
- $f(z_{0})=b_{2}(z_{0})$ and $f(q^{2}z_{0})=b_{2}(q^{2}z _{0})$;
- $f(z_{0})=b_{1}(z_{0})$ and $f(q^{2}z_{0})=b_{1}(q^{2}z _{0})$.

We denote the subset of *B* satisfying (a) by $B_{a}$; similarly, $B_{b}$, $B_{c}$, and $B_{d}$ represent the corresponding subsets of *B*. (28) can lead to the following statements:

(i) $N_{B_{a}} ( r, \frac{1}{f-b_{\ast }} ) > S(r,f)$ and $N_{B_{b}} ( r, \frac{1}{f-b_{\ast }} ) > S(r,f)$;
(ii) $N_{B_{c}} ( r, \frac{1}{f-b_{\ast }} ) >S(r,f)$ and $N_{B_{d}} ( r, \frac{1}{f-b_{\ast }} ) > S(r,f)$;
(iii) $N_{B_{a}} ( r, \frac{1}{f-b_{\ast }} ) >S(r,f)$ and $N_{B\setminus B _{a}} ( r, \frac{1}{f-b_{\ast }} ) = S(r,f)$;
(iv) $N_{B_{b}} ( r, \frac{1}{f-b_{\ast }} ) > S(r,f)$ and $N_{B\setminus B_{b}} ( r, \frac{1}{f-b_{ \ast }} ) = S(r,f)$.

We rewrite (15) as follows:

29 $$\begin{aligned} f(qz)f(z) +f(z)f(z/q) +f(qz)f(z/q) = \frac{\alpha_{4}f^{3} +\alpha_{3}f^{2} +\alpha_{2}f +\alpha _{1} - (\beta_{2}f^{3} +\beta_{1}f^{2})}{f^{2} +\beta_{2}f + \beta_{1}}, \end{aligned}$$

where

$$\begin{gathered} \alpha_{4}=-\bigl(a_{1}(z)+a_{2}(z)+a_{3}(z)+a_{4}(z) \bigr), \\ \alpha_{3}=a_{1}(z)a_{2}(z)+a_{1}(z)a_{3}(z)+a_{1}(z)a_{4}(z)+a_{2}(z)a _{3}(z)+a_{2}(z)a_{4}(z)+a_{3}(z)a_{4}(z), \\ \alpha_{2}=-\bigl(a_{1}(z)a_{2}(z)a_{3}(z)+a_{1}(z)a_{2}(z)a_{4}(z)+a_{2}(z)a _{3}(z)a_{4}(z)\bigr), \\ \alpha_{1}=a_{1}(z)a_{2}(z)a_{3}(z)a_{4}(z), \\ \beta_{2}=-\bigl(b_{1}(z)+b_{2}(z)\bigr), \qquad \beta_{1}=b_{1}(z)b_{2}(z). \end{gathered} $$

Obviously, $\alpha_{i} (i=1,\ldots,4), \beta_{j}(j=1,2)\in S(f)$.

We first consider (i) is valid. For every point $z_{0} \in B_{a}$, substituting $z=qz_{0}$ into (29) yields

30 $$\begin{aligned}& f\bigl(q^{2}z_{0}\bigr)f(qz_{0}) + f(qz_{0})f(z_{0}) + f\bigl(q ^{2}z_{0} \bigr)f(z_{0}) \\& \quad =\frac{(\alpha_{4}(qz_{0}) - \beta_{2}(qz_{0}))f^{3}(qz _{0}) +(\alpha_{3}(qz_{0}) - \beta_{1}(qz_{0}))f^{2}(qz_{0}) + \alpha_{2}(qz_{0})f(qz_{0}) +\alpha_{1}(qz_{0})}{f^{2}(qz_{0}) + \beta_{2}(qz_{0})f(qz_{0}) +\beta_{1}(qz_{0})}. \\& \end{aligned}$$

Note that $f(z_{0})=b_{1}(z_{0})$, $f(qz_{0})=\infty $, and $f(q ^{2}z_{0})=b_{2}(q^{2}z_{0})$, thus comparing the degrees of equation (30), we have

$$ b_{2}\bigl(q^{2}z_{0}\bigr)+b_{1}(z_{0})= \alpha_{4}(qz_{0})-\beta_{2}(qz_{0}) $$

and

$$ b_{2}\bigl(q^{2}z_{0}\bigr)b_{1}(z_{0})= \alpha_{3}(qz_{0})-\beta_{1}(qz_{0})- \beta_{2}(qz_{0}) \bigl(b_{2}\bigl(q^{2}z_{0} \bigr)+b_{1}(z_{0})\bigr). $$

We claim that

31 $$ b_{2}\bigl(q^{2}z\bigr)+b_{1}(z)= \alpha_{4}(qz)-\beta_{2}(qz) $$

and

32 $$ b_{2}\bigl(q^{2}z\bigr)b_{1}(z)= \alpha_{3}(qz)-\beta_{1}(qz)-\beta_{2}(qz) \bigl(b _{2}\bigl(q^{2}z\bigr)+b_{1}(z)\bigr) $$

for all $z \in C$.

Otherwise, it is easy to see that the point $z_{0} \in B_{a}$ must solve equations (31) and (32). By the assumption of (i), we have

$$\begin{aligned} S(r,f) &< N \biggl( \bar{q}^{2}r, \frac{1}{b_{2}(q^{2}z)b_{1}(z)-( \alpha_{3}(qz)-\beta_{1}(qz)-\beta_{2}(qz)(b_{2}(q^{2}z)+b_{1}(z)))} \biggr) \\ & \quad {}+ N \biggl( \bar{q}^{2}r, \frac{1}{(b_{2}(q^{2}z)+b_{1}(z))-( \alpha_{4}(qz)-\beta_{2}(qz))} \biggr) \\ &= S(r,f), \end{aligned} $$

which is a contradiction, thus (31) and (32) are established.

Similarly, it follows from $N_{B_{b}} ( r, \frac{1}{f-b_{\ast }} ) > S(r,f)$ that

33 $$ b_{1}\bigl(q^{2}z\bigr)+b_{2}(z)= \alpha_{4}(qz)-\beta_{2}(qz) $$

and

34 $$ b_{1}\bigl(q^{2}z\bigr)b_{2}(z)= \alpha_{3}(qz)-\beta_{1}(qz)-\beta_{2}(qz) \bigl(b _{1}\bigl(q^{2}z\bigr)+b_{2}(z)\bigr). $$

From (31)–(33), we obtain

35 $$ b_{1}\bigl(q^{2}z\bigr)+b_{2}(z)=b_{2} \bigl(q^{2}z\bigr)+b_{1}(z) $$

and

36 $$ b_{1}\bigl(q^{2}z\bigr)b_{2}(z)=b_{2} \bigl(q^{2}z\bigr)b_{1}(z). $$

Due to $b_{1}(z)$ and $b_{2}(z)$ being distinct functions, we have $b_{1}(q^{2}z)=b_{1}(z)$ and $b_{2}(q^{2}z)=b_{2}(z)$.

Suppose that (ii) is valid. Using the same method as in the case of (31) and (32), we have

37 $$\begin{aligned}& b_{1}\bigl(q^{2}z\bigr)+b_{1}(z)= \alpha_{4}(qz)-\beta_{2}(qz), \end{aligned}$$

38 $$\begin{aligned}& b_{1}\bigl(q^{2}z\bigr)b_{1}(z)= \alpha_{3}(qz)-\beta_{1}(qz)-\beta_{2}(qz) \bigl(b _{1}\bigl(q^{2}z\bigr)+b_{1}(z)\bigr); \end{aligned}$$

39 $$\begin{aligned}& b_{2}\bigl(q^{2}z\bigr)+b_{2}(z)= \alpha_{4}(qz)-\beta_{2}(qz), \end{aligned}$$

40 $$\begin{aligned}& b_{2}\bigl(q^{2}z\bigr)b_{2}(z)= \alpha_{3}(qz)-\beta_{1}(qz)-\beta_{2}(qz) \bigl(b _{2}\bigl(q^{2}z\bigr)+b_{2}(z)\bigr). \end{aligned}$$

Combining (37)–(40) yields $b_{1}(q^{2}z)+b_{1}(z)=b _{2}(q^{2}z)+b_{2}(z)$ and $b_{1}(q^{2}z)b_{1}(z)= [4]b_{2}(q^{2}z)b_{2}(z)$. From $b_{1}(z)$ and $b_{2}(z)$ are distinct, it follows that $b_{1}(q^{2}z)=b_{2}(z)$ and $b_{2}(q^{2}z)=b_{1}(z)$

Suppose that case (iii) holds. By $N_{B_{a}} ( r, \frac{1}{f-b _{\ast }} ) > S(r,f)$, we can also get (31) and (32). Let us define a meromorphic function

41 $$ g(z):=\bigl(f(z)-b_{1}(z)\bigr) \bigl(f(qz)-b_{2}(qz) \bigr). $$

It is easy to see that the poles of $f(z)$ are exactly the zeros of $f(qz)-b_{2}(qz)=0$ and the poles of $f(qz)$ are exactly the zeros of $f(z)-b_{1}(z)=0$ in the set of $B_{a}$. Thus, the poles of $f(z)$ and $f(qz)$ in $B_{a}$ are not the poles of $g(z)$. Therefore, the poles of $g(z)$ in *B* may occur in the complement set of $B_{a}$ or from the poles of $b_{1}(z)$ and $b_{2}(qz)$. By the assumption of (iii), it follows

42 $$ N_{B}(r,g)=S(r,f). $$

From (41), we get

43 $$ f(qz)=\frac{g(z)}{f(z)-b_{1}(z)}+b_{2}(qz),\qquad f(z/q)= \frac{g(z/q)}{f(z)-b _{2}(z)}+b_{1}(z/q). $$

Substituting (43) into (30) yields

44 $$ a(z)f^{2}(z)+b(z)f(z)+c(z)=0, $$

where

$$\begin{aligned}& a(z)=g(z)+g(z/q), \\& \begin{aligned} b(z)&=\beta_{2}(z) \bigl(\alpha_{3}(z)-2 \beta_{1}(z)-\beta_{2}(z)\alpha _{4}(z)+ \beta_{2}^{2}(z)\bigr)+\beta_{1}(z) \alpha_{4}(z) \\ &\quad {}+g(z) \bigl(b_{1}(z/q)-b_{2}(z)\bigr)+g(z/q) \bigl(b_{2}(qz)-b_{1}(z)\bigr)- \alpha_{2}(z), \end{aligned} \\& \begin{aligned} c(z)&=g(z)g(z/q)+\beta_{1}(z) \bigl( \alpha_{3}(z)-\beta_{1}(z)-\beta_{2}(z) \alpha_{4}(z)+\beta_{2}^{2}(z)\bigr) \\ &\quad {}-g(z)b_{2}(z)b_{1}(z/q)-g(z/q)b_{1}(z)b_{2}(qz)- \alpha _{1}(z). \end{aligned} \end{aligned}$$

If $a(z)\not \equiv 0$, $b(z)\not \equiv 0$, $c(z)\not \equiv 0$, then from the definition of $\alpha_{i}$, $\beta_{j}$ ($i=1,\ldots,4$; $j=1,2$) and equation (42), we obtain

45 $$ N_{B}\bigl(r,a(z)\bigr)=S(r,f),\qquad N_{B} \bigl(r,b(z)\bigr)=S(r,f),\qquad N_{B}\bigl(r,c(z)\bigr)=S(r,f). $$

Let us rewrite equation (44) as

46 $$ a(z)f^{2}(z)=-\bigl(b(z)f(z)+c(z)\bigr), $$

then it follows

$$\begin{aligned} N_{B}\bigl(r,a(z)f^{2}(z) \bigr)=2N_{B}(r,f)+S(r,f) \end{aligned}$$

and

$$\begin{aligned} N_{B}\bigl(r,-\bigl(b(z)f(z)+c(z)\bigr) \bigr)=N_{B}(r,f)+S(r,f), \end{aligned}$$

which implies

47 $$\begin{aligned} N_{B}(r,f)=S(r,f), \end{aligned}$$

a contradiction again. Therefore $a(z)=b(z)=c(z)\equiv 0$, which leads to

$$ g(z)=\frac{\beta_{2}(z)(\alpha_{3}(z)-2\beta_{1}(z)-\beta_{2}(z)\alpha _{4}(z)+\beta_{2}^{2}(z))+\beta_{1}(z)\alpha_{4}(z)-\alpha_{2}(z)}{(b _{2}(qz)-b_{1}(z))-(b_{1}(z/q)-b_{2}(z))}. $$

It is obvious that $g(z)\in S(f)$, thus $f(z)$ satisfies the following *q*-difference Riccati equation:

48 $$ f(qz)=\frac{b_{2}(qz)f(z)+g(z)-b_{1}(z)b_{2}(qz)}{f(z)-b_{1}(z)}. $$

Suppose that case (iv) holds. In the same way as case (iii), we can also get $f(z)$ satisfies the *q*-difference Riccati equation

49 $$ f(qz)=\frac{b_{1}(qz)f(z)+g(z)-b_{2}(z)b_{1}(qz)}{f(z)-b_{2}(z)}, $$

where $g(z)$ is a small function related to $f(z)$. The proof of Theorem 3.2 is completed. □
